# The Role of Protein S-Nitrosylation in Mitochondrial Quality Control in Central Nervous System Diseases

**DOI:** 10.14336/AD.2024.0099

**Published:** 2025-03-25

**Authors:** Fang Qiu, Yuqiang Liu, Zhiheng Liu

**Affiliations:** ^1^Department of Burn and Plastic Surgery, Shenzhen Longhua District Central Hospital, Shenzhen, Guangdong, China.; ^2^Department of Anesthesiology, Shenzhen Second People's Hospital, the First Affiliated Hospital of Shenzhen University, Shenzhen, China.

**Keywords:** nitric oxide, protein S-nitrosylation, mitochondrial quality control, central nervous system diseases

## Abstract

S-Nitrosylation is a reversible covalent post-translational modification. Under physiological conditions, S-nitrosylation plays a dynamic role in a wide range of biological processes by regulating the function of substrate proteins. Like other post-translational modifications, S-nitrosylation can affect protein conformation, activity, localization, aggregation, and protein interactions. Aberrant S-nitrosylation can lead to protein misfolding, mitochondrial fragmentation, synaptic damage, and autophagy. Mitochondria are essential organelles in energy production, metabolite biosynthesis, cell death, and immune responses, among other processes. Mitochondrial dysfunction can result in cell death and has been implicated in the development of many human diseases. Recent evidence suggests that S-nitrosylation and mitochondrial dysfunction are important modulators of the progression of several diseases. In this review, we highlight recent findings regarding the aberrant S- nitrosylation of mitochondrial proteins that regulate mitochondrial biosynthesis, fission and fusion, and autophagy. Specifically, we discuss the mechanisms by which S-nitrosylated mitochondrial proteins exercise mitochondrial quality control under pathological conditions, thereby influencing disease. A better understanding of these pathological events may provide novel therapeutic targets to mitigate the development of neurological diseases.

## 1. Introduction

S-Nitrosylation (SNO) refers to a reversible covalent post-translational modification that generates S-nitrosothiols *via* the addition of a nitrosyl group (nitric oxide [NO]) to the sulfhydryl group of cysteine [1]. More than 3,000 proteins are regulated *via* this modification. SNO can alter the spatial conformation of proteins, affect protein intermolecule interactions, and regulate further post-translational modifications, including acetylation, phosphorylation, ubiquitination, and palmitoylation [2, 3]. The common types of post-translational modifications are listed in Table 1. Like phosphorylation, SNO is an important regulator of signal transduction pathways under physiological conditions. However, under pathological conditions, excess NO effectively forces the SNO of cysteine thiols; this abnormal protein SNO leads to protein misfolding, mitochondrial dysfunction, transcriptional dysregulation, synaptic damage, and neuronal injury [4, 5]. Nitrosative stress-mediated excitotoxicity is involved in many neurological disorders including acute ischemic and neurodegenerative diseases.

Mitochondria are essential organelles for a variety of biological processes, including energy production, metabolite biosynthesis, cell death, and immune responses [6]. Mitochondrial dysfunction can result in cell death and has been implicated in the development of many human diseases. Mitochondrial quality control involves mitochondrial biogenesis, mitochondrial dynamics (primarily fusion and fission), and mitochondrial autophagy (mitophagy) [7]. Alterations in mitochondrial quality control are important contributors to the development of a variety of diseases, including neurodegenerative disorders, cancer, and eye diseases [8]. Accordingly, the modulation of mitochondrial quality control may represent an effective strategy for the treatment of these conditions. Although more and more SNO-modifiable mitochondrial proteins are being identified, their roles in mitochondria are not fully understood, nor are the underlying mechanisms.

**Table 1:** List of common types of post-translational modifications.

Name of post-translational modifications	Amino acid residues targeted	Function
**Phosphorylation**	Ser, Thr, Tyr, His, Pro, Arg, Asp and Cys	Protein activation/inactivation, function, folding, signaling, and subcellular localization
**Acetylation**	Lys, Ala, Arg, Asp, Cys, Gly, Glu, Met, Pro, Ser, Thr and Val	Transcriptional activity, protein stability, gene expression, and apoptosis
**Ubiquitylation**	Lys	Protein degradation, subcellular localization, and kinase activation
**Methylation**	Lys, Arg, Ala, Asn, Asp, Cys, Gly, Glu, Gln, His, Leu, Met, Phe and Pro	Gene expression, and chromatin structure
**Glycosylation**	Trp, Ala, Arg, Asn, Asp, Ile, Lys, Ser, Thr, Val, Glu, Pro, Tyr, Cys and Gly	Protein folding, protein structure, transport, cell metabolism, and extracellular interactions
**SUMOylation**	Lys	Nuclear transport, cell cycle, transcriptional regulation, apoptosis, and protein stability
**Palmitoylation**	Cys, Gly, Ser, Thr and Lys	Protein localization, accumulation, secretion, function, and signaling
**Nitrosylation**	Cys	Protein activity, stability, localization, protein-protein interactions, and signaling
**Myristoylation**	Gly and Lys	Plasma targeting, subcellular tracking, localization, function, and signaling
**Prenylation**	Cys	Protein-protein interactions, localization, cellular activity, and trafficking
**Sulfation**	Tyr, Cys, and Ser	Chemical defense, hormone biosynthesis, bioactivation, and steroid biosynthesis
**Citrullination**	Arg	Apoptosis, embryo development, immune system, skin keratinization, insulation, and plasticity
**Crotonylation**	Lys	Gene expression, spermatogenesis, and cell cycle
**Lactylation**	Lys	Cellular metabolism, transcription, and protein interaction
**Hydroxylation**	Pro and Lys	Cell differentiation and proliferation, cell metabolism
**PARylation**	Glu and Asp	DNA repair, chromatin reorganization, transcriptional regulation, apoptosis, and mitosis
**Lipidation**	Cys, Ser, and Lys	Protein conformation, stability, membrane association, localization, trafficking, and binding
**Succinylation**	Lys	Mitochondrial metabolism, cellular respiration, regulation, repair, and tumorigenesis

In this review, we summarize the mitochondrial proteins that are known to undergo SNO, the effects of the aberrant SNO of these proteins on mitochondrial quality control, and the regulatory role of SNO in related diseases.

## Production and detection of S-nitrosylation

NO acts as a messenger molecule in a variety of processes, such as vasodilation and neurotransmission, and also possesses antitumor and antipathogenic activities; however, sustained NO producion can lead to direct tissue toxicity, septic shock, cancer, and inflammation. NO is produced via NOS-mediated oxidation of L-arginine to l-citrulline [9]. NOS can be categorized into four subtypes-neuronal NOS (nNOS), inducible NOS (iNOS), endothelial NOS (eNOS), and mitochondrial NOS (mtNOS) [9, 10]. These isoforms of NOS differ in their localization, regulation, metabolic properties, and inhibitor sensitivity. Although all NOS family members catalyze the production of NO, different NOS isoforms may play distinct regulatory roles during the same pathological or physiological process. nNOS, a calcium-dependent enzyme, is predominantly expressed in peripheral and central neurons and is important for cell-cell communication [11]. In the brain, NO production by neurons involves an important molecular mechanism, namely, the inward flow of Ca^2+^ through activated N-methyl-D-aspartate receptors (NMDARs)[12]. Unlike nNOS and eNOS, iNOS is not constitutively expressed in cells and tissues, instead being induced following cytokines or lipopolysaccharide stimulation [13]. The main function of iNOS is to mediate immune activation, the release of inflammatory mediators, and cell death. eNOS, expressed in blood vessel endothelial cells, plays a key role in the regulation and maintenance of the cardiovascular system [14]. Meanwhile, mtNOS is involved in the regulation of mitochondrial oxygen consumption and mitochondrial transmembrane potential [15]. Although the existence of these NOSs is well established, it is not clear whether other NOS subtypes exist and are active in mammals.

Protein SNO, a post-translational redox modification of cysteine residues, is a non-classical NO signaling mechanism and one of the main means *via* which NO exerts its biological activity [4]. In addition, NO can be transferred between thiol groups of different proteins *via* a transnitrosylation reaction [16, 17]. SNO occurs only at specific cysteine residues within proteins, and this specificity depends on the following conditions: First, the target cysteine must be close to the source of NO production to increase the likelihood of SNO occurring [18]. Secondly, the target cysteine must lie within a specific motif—I/L-X-C-X2-D/E—that can be recognized by NOS [19]. Thirdly, the target cysteine should preferentially be in the right environmental context, such as an appropriate pH [20]. Finally, the target cysteine should be within a highly hydrophobic environment, such as that resulting from tertiary protein structures or within membranes [21]. SNO is highly regulated in both time and space, and its reversibility makes it a versatile mechanism through which NO can control gene expression programs in health and disease.

**Figure 1.**
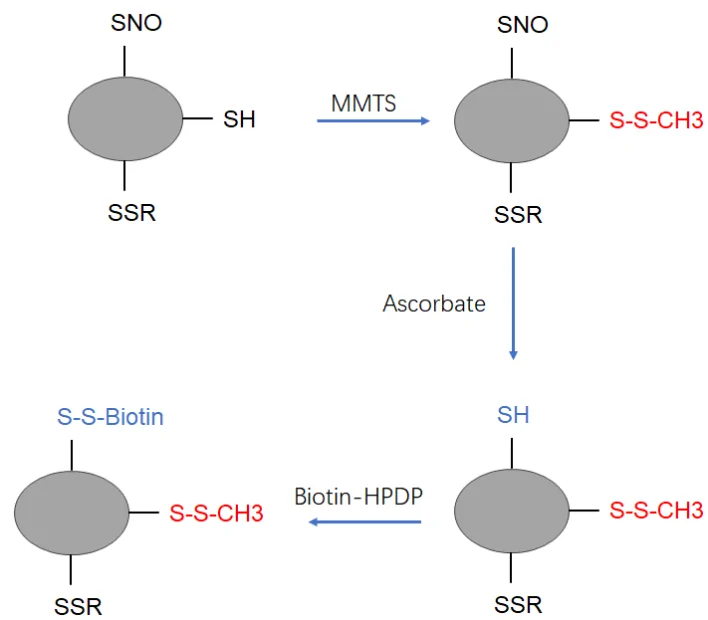
**Schematic representation of the biotin switch assay**. Proteins extracted from tissue or cells are incubated with methyl methanethiosulfonate (MMTS), which blocks all free thiols. Unreacted MMTS is removed by acetone precipitation. After blocking, ascorbate is used to reduce S-nitrosothiol bonds. Finally, reduced thiols are labeled using biotin-HPDP.

Although SNO has been identified as an important means of regulating protein function, its reversibility and instability, as well as the low abundance of endogenous SNO, have limited its research applications. The most commonly used indirect method for SNO detection is the biotin switch assay (BSA), pioneered by Jaffrey et al [22]. The BSA involves replacing nitrosylated cysteine residues with biotinylated ones and then performing a western blot with anti-biotin antibodies to detect the biotinylated proteins (Fig. 1). Several methods based on variations of the BSA have since emerged that have improved SNO quantification [23]. With the rapid advances in proteomic techniques, mass spectrometry coupled with some improved BSA-based techniques has enabled the rapid development of SNO proteomics research [24-27].

SNO affects protein structure and function by modulating enzymatic activities, protein-protein interactions, and protein subcellular localization, and also influences a variety of cellular processed, including protein stability, transcriptional regulation, epigenetic regulation, cellular energy metabolism, metal transport and homeostasis, protein translation and post-translational modifications, protein degradation, DNA damage repair, apoptosis, and redox regulation [28-30]. Thus, SNO is an emerging paradigm for signal transduction and the modulation of protein properties. However, dysregulated SNO is associated with many human diseases involving mitochondrial dysfunction, cell death, and metabolic reprogramming, among other processes.

**Figure 2.**
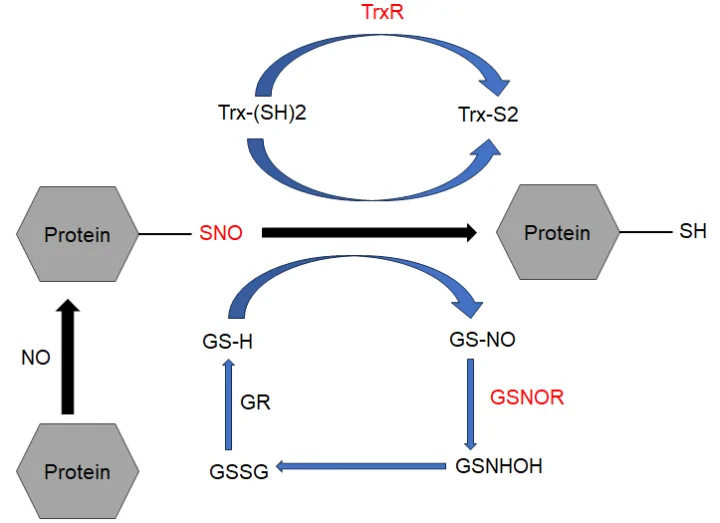
**The mechanisms that mediate protein denitrosylation**. Two major enzymatic systems mediate the denitrosylation of proteins. One is the Trx/TrxR system, which mediates denitrosylation through the reduction of the S-nitrosothiol groups of proteins. The other is the GSNOR/GR kinase system, whereby GSNOR converts GSNO to glutathione S-hydroxysulfenamide (GSNHOH) *via* the reducing power of NADH; subsequently, GSNHOH is converted to oxidized glutathione (GSSG), which is then reduced to glutathione (GSH) by glutathione reductase (GR), resulting in denitrosylation.

Importantly, SNO is usually a non-enzymatic reaction, whereas denitrosylation can be either enzymatic or non-enzymatic [31]. The breaking down of S-N bond of SNO can occur in the presence of factors such as reducing agents, UV, metal ions, and heat. S-nitrosocysteine (SNOC) and S-nitrosoglutathione (GSNO) are common NO donors for the generation S-nitrosothiols in cells. They can release NO under physiological conditions and are used to mimic the biological effects of endogenous NO, acting via the sGC-cGMP pathway [9]. Accordingly, they are often employed in *in vitro* experiments to probe the molecular mechanisms underlying the pathological effects of NO-mediated nitrosative stress in vivo. SNO levels are modulated by several mechanisms, one of which is denitrosylation, a process that is regulated by the S-nitrosoglutathione reductase (GSNOR) and the thioredoxin (Trx) systems (Fig. 2) [32]. It is becoming increasingly clear that SNO and denitrosylation are precisely regulated both temporally and spatially. The balance between nitrosylation and denitrosylation determines the extent of protein nitrosylation, which ensures proper cellular homeostasis. The disruption of this balance can lead to a wide range of diseases [33].

## S-Nitrosylation of mitochondrial proteins

Mitochondria are a key site of reactive oxygen species (ROS) production [34]. Mitochondrial proteins become S-nitrosylated in response to changes in mitochondrial respiration and redox imbalance. This protects protein thiols and cells from oxidative damage, while also preventing further ROS generation [35, 36]. SNO and denitrosylation play an integral role in mitochondrial bioactivity and quality control [37, 38]. Recent findings relating to S-nitrosylated mitochondrial proteins in brain disorders are listed in Table 2. It was recently reported that 56% of S-nitrosylated proteins in the heart are mitochondrial proteins [39]. In addition, mitochondrial proteins were shown to account for 52% of all S-nitrosylated proteins in cardiac tissue of mice after ischemic preconditioning [33]. The incubation of rat heart mitochondrial lysates withGSNO, led to the identification of more than 60 S- nitrosylated proteins that are involved in solute transport, electron transfer, and the TCA cycle, among other processes [35].

**Figure 3.**
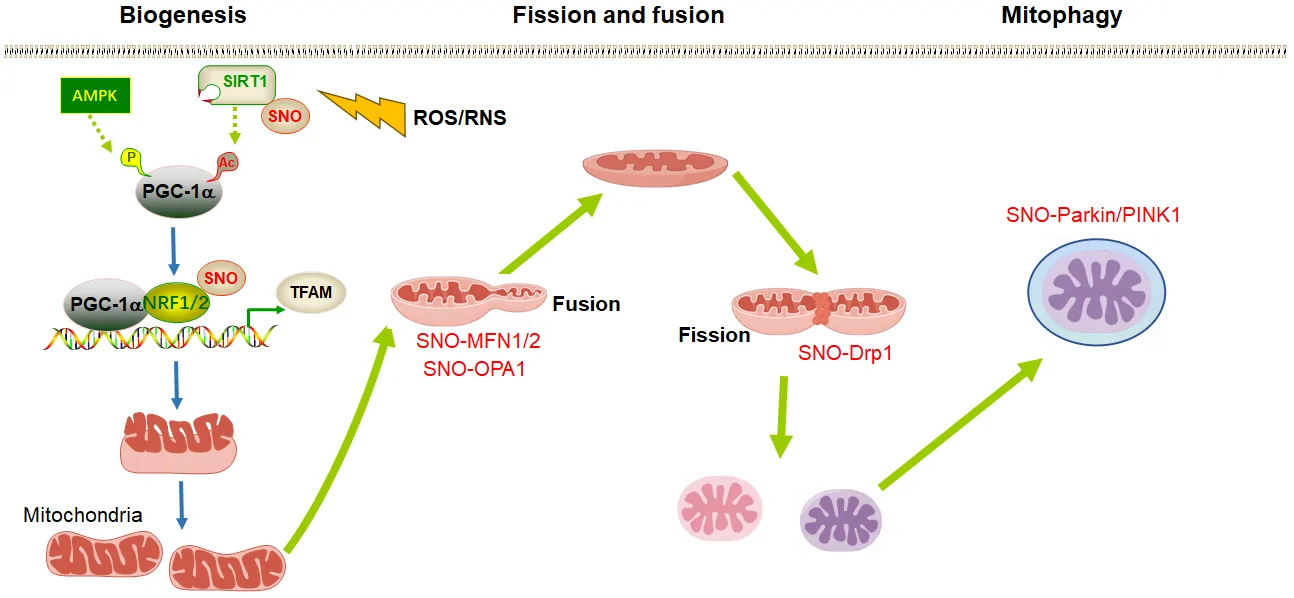
**The regulation of mitochondrial quality control by protein S-nitrosylation**. Mitochondrial quality control regulates mitochondrial biogenesis, mitochondrial fission and fusion, and mitochondrial autophagy. When the reactive oxygen species (ROS)/reactive nitrogen species (RNS) balance is disturbed, nitric oxide (NO) production and protein S-nitrosylation are abnormally increased. NO-mediated S-nitrosylation affects mitochondrial function mainly *via* the S-nitrosylation of key proteins involved in the three above-mentioned processes, which leads to synapse loss and neuronal death.

SNO acts on mitochondrial quality control by regulating mitochondrial biogenesis, mitochondrial dynamics, and mitophagy via Drp1, PTEN-induced kinase 1 (PINK1), and Parkin RBR E3 ubiquitin-protein ligase (Parkin) (Fig. 3). Additionally, SNO regulates mitochondrial metabolism through its effects on proteins related to the TCA cycle, fatty acid oxidation, amino acid metabolism, and ketolysis [63]. All mitochondrial complexes can be S-nitrosylated and, consequently, inhibited, thereby minimizing ROS production under oxidative conditions [57]. Similarly, all proteins involved in the TCA cycle can be modified by nitrosylation [53]. The effect of SNO on these proteins is inhibitory, decreasing the production of reduced nicotinamide adenine dinucleotide (NADH). In contrast to these inhibitory effects, the SNO of Cys501 of the mitochondrial chaperone TRAP1 in hepatocellular carcinoma cells was shown to promote TRAP1 degradation, thereby increasing succinate dehydrogenase (SDH) levels and activity [69].

**Table 2:** List of proteins regulated by S-nitrosylation in mitochondria.

Protein	Cysteine residue	Effect of S-nitrosylation	References
**Drp1**	644	Increased Drp1 enzymatic activity, excessive mitochondrial fragmentation, synapse impairment, neuronal cell death	[40-42]
**PINK1**	568	Inhibition of PINK1 kinase activity, reduced phosphorylation levels, enhanced Parkin phosphorylation, inhibition of mitophagy	[43, 44]
**Parkin**	241, 260, 323	Regulation of Parkin E3 ubiquitin ligase activity and mitophagy, increased Drp1 expression and activity	[38, 44, 45]
**OPA1**	Unknown	Unknown	[46]
**VDAC1**	232	Neuronal death and synaptic dysfunction	[47, 48]
**VDAC2**	47, 62, 91, 92, 118	Mitochondrial permeability transition pore opening and cell death	[47-49]
**HSP60**	442, 237	Facilitated interactions with proteins required for maintaining mitochondrial DNA stability, mediated the effect of statins on endothelial integrity, regulation of mitochondrial energy production	[47, 50]
**ACAT1**	119, 410	Inhibition of ACAT1 activity, decreased acetylcholine production	[47, 51]
**ACSL3**	450, 503	Reduced lipid synthesis, fatty acid degradation	[47, 52]
**ACSL4**	221, 503	Reduced lipid synthesis, fatty acid degradation	[47, 52]
**GAPDH**	149, 150, 152	Inhibition of glycolysis and regulation of cell death	[53]
**Complex I**	39	Inhibition of mitochondrial respiration	[54, 55]
**Complex II**	536	Inhibition of mitochondrial respiration	[36, 55, 56]
**Complex III**	268	Inhibition of mitochondrial respiration	[55, 56]
**Complex IV**	196, 200	Inhibition of mitochondrial respiration	[55, 57]
**Complex V**	244, 294	Inhibition of its ATPase activity	[55, 58]
**ACO**	12	Suppression of the citric acid cycle	[59, 60]
**SOD1**	111	Inhibition of its monomerization and inhibition of cell apoptosis	[61]
**SOD2**	8	Inhibition of its detoxifying capacity	[39, 48]
**PRDX2**	51,172	Inhibited its activity, increased oxidative stress	[62]
**IDH**	148, 297, 336, 379, 418	Suppressed the conversion of isocitrate to α-ketoglutarate	[39, 53]
**α-KGDH**	Unknown	Regulation of its activity	[39, 53, 59, 60]
**SCS**	Unknown	Regulation of succinylation	[36, 53]
**GDH**	112	Inhibited its activity	[39, 53, 63]
**CS**	101	Increased its activity	[39, 53, 64]
**MDH**	89, 125, 516	Enhanced its activity	[39, 53, 64-66]
**VLCAD**	238	Enhanced its catalytic activity	[39]
**HK**	108	Inhibited its activity	[53, 67]
**ALDOC**	202	Inhibited its activity	[39, 48]
**PDH**	43	Inhibited its activity	[39, 63, 68]

Abbreviations: Drp1, dynamin-related protein 1; PINK1, PTEN induced kinase 1; OPA1, optic atrophy protein 1; VDAC, voltage-dependent anion channel; HSP60, heat shock protein 60; ACAT1, acetyl-CoA acetyltransferase 1; ACSL, long-chain-fatty-acid-CoA ligase; GAPDH, glyceraldehyde-3-phosphate dehydrogenase; ACO, aconitase; SOD, superoxide dismutase; PRDX2, peroxiredoxin-2; IDH, isocitrate dehydrogenase; α-KGDH, α-ketoglutarate dehydrogenase; SCS, succinyl-CoA synthetase; CS, citrate synthase; GDH, glutamate dehydrogenase; MDH, malate dehydrogenase; VLCAD, very long chain acyl-CoA dehydrogenase; HK, hexokinase; ALDOC, fructose-bisphosphate aldolase C; PDH, pyruvate dehydrogenase.

As mentioned above, SNO, in addition to preventing ROS production and protecting protein thiols from oxidative damage, also protects cells from death. The latter effect may be explained by the fact that reduced energy production induces cell death, and inhibiting the cell death pathway may represent a counteracting mechanism to reduced energy levels. Pro- and anti-apoptotic proteins known to be S-nitrosylated include the mitochondrial permeability transition pore (mPTP), voltage-dependent anion channels (VDACs), caspases, and Bcl-2. Under physiological conditions, SNO inhibits mPTP formation, whereas, under pathological conditions, aberrant SNO activates the mPTP and promotes apoptosis [70]. VDACs play a major role in mitochondria-mediated apoptosis by regulating cytochrome c release [71]. Like mPTP, the effect of SNO on VDACs has been reported to be biphasic [72], that is, low concentrations of NO inhibit VDAC function and prevent the activation of apoptosis, while high concentrations of NO elicit the opposite effect. The SNO of caspase or Bcl-2 exerts anti-apoptotic effects.

## The effect of SNO on mitochondrial quality control

### The regulation of mitochondrial biogenesis by SNO

Mitochondrial biogenesis is a process by which a cell produces new mitochondria and involves an increase in size and the subsequent division of the organelles. Mitochondrial biogenesis is mainly regulated by the transcriptional co-activator peroxisome proliferator-activated receptor gamma coactivator 1-alpha (PGC-1α) [73]. The phosphorylation (elevated Ca^2+^ and AMP levels) or deacetylation (sirtuin 1 [SIRT1]) of PGC-1α leads to its activation, following which it binds to nuclear respiratory factor 1 and 2 (NRF-1 and NRF-2), leading to the expression of a wide range of mitochondrial genes, including mitochondrial transcription factor A (TFAM) [74, 75]. The treatment of dopaminergic (DA) neurons or SH-SY5Y cells with SNOC, a NO donor, was shown to induce Parkin-interacting substrate (PARIS), which contributed to PGC-1α nuclear translocation; this resulted in a reduction in mitochondrial DNA copy number and ATP concentrations, leading to mitochondrial dysfunction [76]. The nuclear SNO of p53 at cysteine C124 promotes the binding of p53 to the -2317 p53RE on the promoter of the mouse *Ppargc1a* gene (encoding PGC-1α), inducing its transcription. Decreased nuclear localization of the nNOS/syntrophin complex was reported to decrease the SNO of p53, which negatively affected the levels of PGC-1α, leading ultimately to changes in mitochondrial biogenesis [77]. Nuclear factor erythroid 2-related factor 2 (Nrf2) is a regulator of the cellular redox status, and thus influences mitochondrial membrane potential, oxidative phosphorylation, and the rate of ATP synthesis [78]. Nrf2 can also modulate mitochondrial biogenesis by upregulating the expression of Nrf1 and PGC-1α [79, 80]. It has been shown that the treatment of rat pheochromocytoma cells with the NO donor S-nitroso-N-acetylpenicillamine (SNAP) induced the SNO of Keap1, which prompted the nuclear translocation and subsequent activation of Nrf2 [81]. Additionally, enzyme HO-1 expression was increased upon upregulation of Nrf2 phosphorylation, which protected cells from death. In this case, the protein SNO may be timely and important for NO-induced cellular nitrosative stress.

### The regulation of mitochondrial dynamics by SNO

Mitochondria are highly dynamic organelles that constantly undergo fission and fusion, leading to the continuous rearrangement of the mitochondrial network. Under normal conditions, fission and fusion are in a state of equilibrium; however, this equilibrium can be disrupted when metabolism or environmental stresses change. Mitochondrial fission is the process whereby one mitochondrion separates into two or more organelles, while fusion refers to the mechanism by which two or more mitochondria fuse into one. Mitochondrial fission is mediated by the GTPase dynamin-related protein 1 (Drp1, Dnm1 in yeast) and cofactors [82, 83]. Mitochondrial fusion, meanwhile, is dominated by the mitochondrial proteins mitofusin (MFN) 1/2 and optic atrophy protein 1 (OPA1), which are localized to the outer and inner mitochondrial membranes, respectively [84, 85]. MFN1/2 mediates fusion of the outer mitochondrial membrane, whereas OPA1 mediates fusion of the inner mitochondrial membrane.

The activity of Drp1 is regulated through post-translational modifications [86]. Phosphorylation at Ser616 activates Drp1, which promotes mitochondrial fission, while phosphorylation of the Ser637 site of Drp1 inhibits its activity [87]. Nitrosative stress leads to the aberrant SNO of Drp1 at Cys644, which increases its enzymatic activity, resulting in excessive mitochondrial fragmentation and synapse impairment [40]. Mutation of the Cys644 site of Drp1, which prevents its nitrosylation, reduces mitochondrial fission, further confirming the importance of the SNO modification of Drp1 in mitochondrial dynamics. In contrast, Bossy et al. [46] reported that the NO-mediated SNO of Drp1 does not affect the GTPase activity of the latter, but instead triggers the phosphorylation of Ser616 of Drp1, leading to its activation and recruitment to mitochondria. Recent studies have shown that increased levels of SNO-Drp1, enhanced Drp1-S616 phosphorylation, and mitochondrial elongation can occur simultaneously [41]. Furthermore, under physiological conditions, the application of Nω-nitro-L-arginine methyl ester hydrochloride (L-NAME), a NOS inhibitor, led to mitochondrial elongation and a decrease in both SNO-Drp1 and Drp1-S616 phosphorylation levels. These findings suggest that SNO-Drp1 regulates Drp1-S616 phosphorylation, thereby promoting mitochondrial fission. In summary, the mechanism underlying the effects of NO on mitochondrial fission requires further in-depth investigation, especially relating to the function of Drp1 and the role of single or multiple simultaneous protein post-translational modifications in this process. Little is known about the effect of SNO on mitochondrial fusion-associated proteins (MFN1/2 and OPA1); additionally, except for OPA1, whether these proteins are S-nitrosylated has not been experimentally confirmed. In an in vivo study, Bossy et al[46]. found that OPA1 can be S-nitrosylated, but the practical meaning of this modification remained unclear. More experiments are needed to elucidate the role of SNO in mitochondrial fusion.

### The regulation of mitophagy by SNO

Mitophagy refers to a process whereby cells selectively encapsulate and degrade damaged or dysfunctional mitochondria in the cell to maintain mitochondrial homeostasis and prevent damage to the cell. There is growing evidence that excessive SNO disrupts mitochondrial autophagy, leading to the accumulation of damaged or dysfunctional mitochondria. In GSNOR-deficient mice, protein SNO, mitophagy, and mitochondrial fragmentation are increased, while autophagic flux is unchanged, both in the liver and the brain [88]. PINK1- and Parkin-mediated mitophagy is an important cellular mechanism underlying the regulation of mitochondrial dynamics [89]. Following mitochondrial damage, the mitochondrial membrane potential decreases, and PINK1 accumulates on the outer mitochondrial membrane, where it recruits and activates Parkin and other autophagy-associated proteins, thereby initiating mitophagy [90].

**Figure 4.**
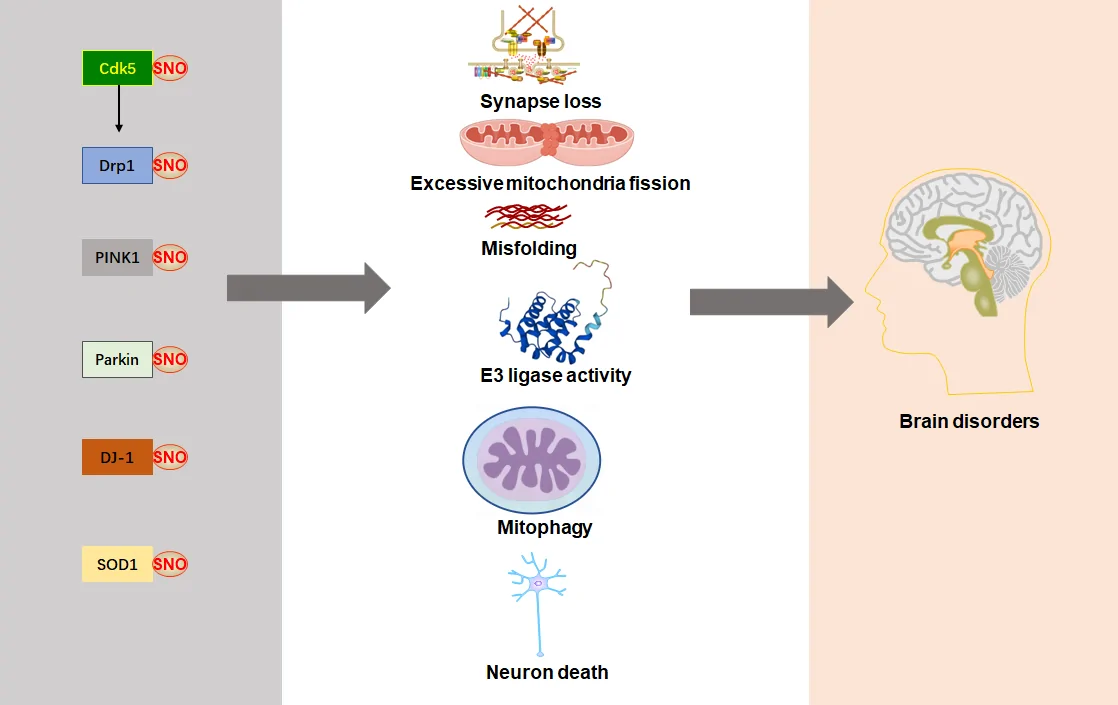
**Neurologic disease triggers aberrant S-nitrosylation reactions**. Aberrantly, S-nitrosylated proteins respond to stimuli in neurological disorders. The overproduction of nitric oxide (NO) can lead to the S-nitrosylation of many proteins, including Cdk5, Drp1, and Parkin. Subsequently, these aberrantly S-nitrosylated proteins can trigger neurotoxic signals, leading to synaptic damage, impaired mitochondrial function, protein misfolding, abnormal E3 ligase-mediated ubiquitination, mitochondrial autophagy, and neuronal death, which further contributes to the pathology of brain disorders.

In the brains of GSNOR knockout mice, the expression of SNO-Parkin is abnormally upregulated, whereas mitochondrial autophagy is inhibited [88]. Aberrant SNO-PINK1 inhibits PINK/Parkin-mediated mitophagy, leading to neuronal death [43, 91]. The SNO of PINK1 at Cys568 inhibits its kinase activity and reduces the level of phosphorylation, which, in turn, enhances downstream Parkin phosphorylation, inhibits Parkin translocation into mitochondria, and ultimately inhibits mitophagy [43, 44]. In addition, one study reported that the NO-induced SNO of Parkin inhibited its E3 ubiquitin ligase activity and reduced the level of Parkin-mediated ubiquitination, which impaired the protective effect of Parkin against environmental toxicity-induced cell death [92]. However, other studies have found that the SNO of Parkin induced by the NO donor GSNO leads to an increase in Parkin E3 ubiquitin ligase activity and promotes mitochondrial degradation [38]. This difference may be due to the time dependence of SNO and denitrosylation regulation. Parkin E3 ubiquitin ligase activity increases after only 3 hours of treatment with a NO donor and decreases after 6 hours. Notably, Parkin SNO and ubiquitination levels at later time points have not been reported to date, for instance, after 12-24 hours of incubation with NO donors. There is also the important issue of the key Parkin cysteine nitrosylation sites. Parkin has multiple RING structural domains, with the IBR structural domain being located between RING1 and RING2 [93]. It was found that the SNO of Cys323, located in the IBR domain of Parkin, increases its E3 ubiquitin ligase activity, and this site is not involved in zinc ion coordination [38]. In addition, treatment with a NO donor induced the auto-ubiquitination of wild-type Parkin, but not the Parkin C323A or Parkin C323S mutants, suggesting that SNO regulates Parkin activity. Five of the seven cysteines in the RING1 structural domain of Parkin were found to be candidate SNO sites using quadrupole time-of-flight mass spectrometry, and the role of these sites in Parkin E3 ubiquitin ligase activity requires further investigation [44].

### Abnormally S-nitrosylated mitochondrial proteins in central nervous system diseases

Many S-nitrosylated proteins respond to stimuli in neurological disorders, leading to protein misfolding and mitochondrial damage, among other effects (Fig. 4). Next, we discuss the effects of the aberrant SNO of mitochondrial proteins on mitochondrial quality control in different brain diseases.

### Alzheimer’s disease

Alzheimer's disease (AD) is a slow-progressing neurodegenerative disorder characterized by the presence of neuroinflammatory plaques and neurogenic fiber tangles in the brain [94]. Age is the greatest known risk factor for AD while memory impairment is its key feature [95, 96]. It has been estimated that 6.7 million Americans aged 65 and older are living with AD, and this number is expected to grow to almost 13 million by 2050. In 2032, the financial caregiving burden for this condition has been estimated to reach $345 billion [97]. Currently, there is no cure for AD, and the available treatments merely improve its symptoms.

Many studies have shown that aberrant protein SNO is associated with the pathogenesis of AD [17, 40, 98, 99]. A study investigating SNO in hippocampal, striatal, and cortical tissues of AD patients using mass spectrometry coupled with proteomic techniques identified 45 endogenously S-nitrosylated proteins, which were found to be mainly involved in metabolism, signaling pathways, apoptosis, and redox regulation [48]. The authors highlighted three important differentially upregulated S-nitrosylated proteins, namely, superoxide dismutase [Mn], mitochondrial (SOD2), voltage-dependent anion-selective channel protein 2 (VDAC2), and fructose-bisphosphate aldolase C (ALDOC). However, the effects of the aberrant SNO of these proteins on mitochondrial quality control and even AD are unclear and need to be explored in detail. Additionally, high-throughput analysis of endogenous SNO in brain homogenates from APP/PS1 AD model mice detected 135 S-nitrosylated proteins, most of which were found to be associated with metabolic and signal transduction pathways [100]. These inconsistencies in differentially nitrosylated proteins may be due to differences in tested samples and detection techniques. Future studies should concentrate on identifying differences in abnormal S-nitrosylated proteins between different cell types (glial cells, neurons) or different brain regions (cortex, striatum) and overall protein samples.

VDACs are present on the outer mitochondrial membrane and play a key role in the regulation of mitochondrial energy metabolism and mitochondria-mediated apoptosis. VDAC1 and VDAC2, but not VDAC3, were found in the brains of AD patients [101]. In addition, total VDAC1 protein content was significantly reduced in the frontal cortex and thalamus of the patients, whereas VDAC2 was markedly elevated only in the temporal cortex. In contrast, other studies have shown that VDAC1 expression is abnormally increased in the brains of AD patients and amyloid precursor protein (APP) transgenic mice [102]. The N-terminal structural domain of VDAC1 can interact with Aβ oligomers, allowing Aβ to enter the cell, which it interacts with the N-terminal structural domain of mitochondrial VDAC1; this leads to the oligomerization of VDAC1, followed by the release of cytochrome c, and, subsequently, apoptosis [103]. Alterations in the levels of these proteins impair neuronal synaptic density and mitochondrial function, thereby influencing the pathology of AD. Although VDAC1 and VDAC2 hypernitrosylation has been reported [48], the extent of this nitrosylation is unknown, as is how hypernitrosylation affects their expression; additionally, VDAC1 and VDAC2 hypernitrosylation may be related to altered mPTP function and oxidative damage in mitochondria. The nitrosylation of VDAC1 can alter the function of its channel, leading to an imbalance in mitochondrial calcium homeostasis and, ultimately, impaired cognition. SNO is known to influence not only protein expression and function but also protein interactions. When S-nitrosylated, VDAC1 can interact with VDAC2, as well as other binding partners, including beta-actin, other cytoskeletal proteins, and pro-apoptotic proteins (such as Bax/Bak). This can result in conformational changes and the oligomerization of VDAC, which leads to the formation of a cytochrome c release channel and the triggering of apoptosis [48, 104, 105]. These observations strongly indicate that therapeutic strategies targeting the nitrosylation of VDAC or its interactions with other proteins may represent potential new approaches for mitigating or preventing the onset or progression of AD.

Cyclin-dependent kinase 5 (Cdk5) is a predominantly neuron-specific kinase that plays a critical role in synaptic plasticity and memory formation. SNO-Cdk5 mediates the Aβ-induced loss of dendritic spines and neuronal damage through the transnitrosylation of Drp1, forming SNO-Drp1, which regulates mitochondrial quality control [17, 98]. Aβ_25-35_ induces mitochondrial fission in a NO-dependent manner, leading to synaptic loss and neuronal damage [106, 107]. Additionally, NO donors induced the formation of SNO-Drp1. The treatment of brain cortical neurons with Aβ25-35 reportedly induced pathologic mitochondrial fragmentation via the SNO of Drp1. Similarly, high levels of SNO-Drp1 were found in the brains of both Tg2576 AD model mice and patients with AD [40]. Notably, the use of a non-nitrosylatable Drp1 mutant (C644A) prevented mitochondrial fission, synaptic loss, and neuronal cell death, suggesting that targeting the SNO of Drp1 at Cys644 may be a promising therapeutic strategy for AD.

### Parkinson’s disease

Parkinson’s disease (PD) is a movement disorder typified by the loss of DA neurons in the substantia nigra and the aggregation of alpha-synuclein in the form of Lewy bodies [108]. PD is the second most common neurodegenerative disorder after AD [109]. NO has been reported to exert both neuroprotective and neurotoxic effects in neurodegenerative diseases [110]. NO-induced oxidative stress and nitrosative stress play a significant role in PD progression [111, 112]. Many aberrantly S-nitrosylated proteins have been implicated in PD [113-115].

Parkin and PINK1 act synergistically to regulate mitochondrial homeostasis by inducing mitochondrial autophagy, thereby promoting cell survival. Parkin induces mitophagy by ubiquitinating a variety of substrates, such as Mfn1/2 on damaged mitochondria [116]. In humans, mutations in the Parkin geneare associated with autosomal recessive familial PD. The levels of SNO-Parkin were found to be significantly elevated in the brains of patients with PD as well as PD model animals [38, 44, 92]. That Parkin is S-nitrosylated raises several questions, such as which are the SNO sites on Parkin; how does activating or inhibiting SNO affect Parkin activity; and how does SNO affect the activities of other E3 ligases/antioxidants, mitochondrial structure/ dynamics, and cell survival. The treatment of HEK293 cells with GSNO for 2 h enhanced the E3 ubiquitin ligase activity of Parkin, whereas treatment for 6 h resulted in the inhibition of Parkin-mediated ubiquitination of synphilin-1 [92, 117]. This suggests that the chronic elevation of nitrosative stress leads to an overall decrease in Parkin E3 ligase activity. Similar phenomena have been found in other cell lines. Lipton et al. reported that treatment with the NO donor SNOC or rotenone increased the E3 ligase activity of Parkin in SH-SY5Y cells within a few hours, but this increase began to be reversed within 12-24 h treatment [44]. In addition, the level of Parkin SNO was reduced compared with that in the blank control group (basal level), and was also lower after rotenone or CCCP treatment [38]. This bidirectional action suggests that the regulation of Parkin E3 ligase activity by SNO/de-nitrosylation is time-dependent, with SNO being predominant in the early stages and de-nitrosylation in the later stages. However, further studies will help to better understand the relationship between the biphasic regulation of the E3 ubiquitin ligase activity of Parkin and the pathogenesis of PD.

Many studies have reported the identification of the Parkin residues that undergo nitrosylation, but the results have been contradictory. Yoshizumi et al. [38] showed that SNO on Cys323 plays a key role in promoting Parkin E3 ligase activity, while other groups identified multiple sites for Parkin nitrosylation that exerted the opposite effects [44, 92]. This discrepancy may be explained by the different sample preparation techniques used in the studies. The former used a combination of Parkin truncations and fixed-point mutations to analyze the nitrosylation of cysteine residues, while in the latter, the authors treated cell lysates with GSNO and recombinant Parkin protein with SNOC. Subsequent studies showed that in Parkin-transfected SH-SY5Y cells, GSNO or 1-methyl-4-phenylpyridinium ion (MPP^+^) treatment for 24 h increased the levels of oxidized NO, leading to the SNO of Parkin. Further analysis revealed that the nitrosylation of the Cys241, Cys260, and Cys323 residues of Parkin inhibited the Parkin-mediated ubiquitination of divalent metal transporter 1 (DMT1) and that these three Cys residues work together to regulate the E3 ubiquitin ligase activity of SNO-Parkin [45]. However, whether Parkin harbors other SNO sites remains unclear. Morever, the levels of nitrosylated Parkin were found to be increased at different time points in the brain tissue of 1-methyl-4-phenyl-1,2,3,6-tetrahydropyridine (MPTP)-injected mice [92], but, the reason for this increase remains to be explained.

It has been shown that the SNO of Parkin alters its nuclear localization, reduces its ability to inhibit p53 expression, increases neuronal death, and promotes the progression of sporadic PD [118]. Parkin function is also regulated by phosphorylation and subsequent recruitment, and it has been shown that SNO and phosphorylation independently regulate Parkin function [38]. A recent study showed that the SNO of parkin resulted in increased Drp1 expression and activity in MPP^+^-treated Parkin-transfected SH-SY5Y cells. In addition, SNO-Drp1 levels were not upregulated in an MPTP-induced mouse model of PD or MPP^+^-treated SH-SY5Y cells [119], suggesting that the SNO of Drp1 does not play an essential role in PD-related mitochondrial dynamics.

Like Parkin, PINK1, is also a key player in mitophagy and is associated with early onset forms of PD [120]. Under stressful conditions, PINK1 undergoes autophosphorylation and dimerization, leading to the accumulation of full-length PINK1 at the outer mitochondrial membrane, the recruitment of Parkin to the damaged mitochondria, and, subsequently, mitophagy [121]. Transgenic mice overexpressing alpha-synuclein, which partially mimics sporadic PD, show elevated levels of SNO-PINK1 as well as defective mitochondrial quality control [43]; in contrast, alpha-synuclein knockout cells and mice are resistant to the deleterious effects of reactive nitrogen species (RNS) generated through exposure to MPP^+^ or lipopolysaccharide [122]. Interestingly, PINK1 SNO, but not Parkin SNO, was detected in the PD model mice, suggesting that PINK1 is more sensitive to SNO, and that its SNO occurs before that of Parkin in sporadic PD pathology. Studies have shown that Cys568 is the main site of SNO-PINK1. The SNO of PINK1 decreases its kinase activity (reduced levels of PINK1 autophosphorylation and Parkin phosphorylation), reduces the expression of full-length PINK1, inhibits Parkin translocation to mitochondria, impairs mitophagy, and increases cell death. Studies using human stem cell-derived DA neurons have demonstrated that neurons carrying the endogenous alpha-synuclein-A53T mutation show increased kinetics of NO synthesis and are more susceptible to nitrosative stress [123, 124]. Taken together, these observations indicate that alpha-synuclein aggregation/nitrosative stress interactions contribute to the progression of PD.

The loss of DJ-1 functions, which leads to neurodegeneration, is closely associated with PD [125]. DJ-1 complements aspects of the PINK1/Parkin mitophagy pathway [126, 127]. DJ-1 regulates the SNO of Parkin and other proteins through transnitrosylation [128], suggesting that SNO-DJ-1 interferes with mitophagy by inactivating the E3 ligase activity of Parkin through transnitrosylation. This may be important for the NO-mediated regulation of PD pathogenesis.

### Huntington’s disease

Huntington’s disease (HD) is an autosomal dominant neurodegenerative disorder that typically results in motor, cognitive, and psychiatric disturbances [129]. The main cause of the disease is an abnormally amplified CAG repeat sequence near the N-terminus of the Huntington gene (*HTT*), which results in the production of a mutant Huntington protein (mHTT). Although evidence of a direct association between NO and HD is lacking, substantial evidence supports that abnormally S-nitrosylated proteins are involved in the pathogenesis of HD. Norris and colleagues [130] reported that nNOS mRNA levels are reduced in the striatum and dorsal caudate nucleus, but not the cortex, of patients with HD. In addition, both nNOS activity and nNOS protein expression were found to be reduced in the R6/2 transgenic mouse model of HD [131]. This suggests that reduced nNOS activity and expression contribute to increased oxidative stress in HD.

Previously, we mentioned that the formation of SNO-Drp1 in AD increases the GTPase activity of Drp1 and leads to mitochondrial fragmentation, which impairs bioenergetics and induces synaptic damage and neuronal loss. Similarly, abnormal mitochondrial dysfunction is also a characteristic of HD, and SNO-Drp1 formation has also been reported to influence HD pathology. The levels of SNO-Drp1 were found to be elevated in the striatum of transgenic HD model mice as well as in the postmortem brains of patients with the disease [42]. The abnormal SNO of Drp1 induces excessive mitochondrial fragmentation with a subsequent loss of dendritic spines. However, these phenotypic changes can be reversed by transfection with the non-nitrosylatable mutant of Drp1 (C644A) or by blocking NO production with a NOS inhibitor. Furthermore, SNO-Drp1 levels were increased in a brain region (striatum) that displays neurotoxicity in the early stages of HD, suggesting that SNO-Drp1 initiates mitochondrial fragmentation and synaptic damage in the early stages of the disease. The results of this study suggested that SNO-Drp1 may be a novel therapeutic target for alleviating synaptic and mitochondrial damage in HD.

### Amyotrophic Lateral Sclerosis

Amyotrophic Lateral Sclerosis (ALS), also known as motor neuron disease, is a devastating neurodegenerative disorder [132]. The incidence of ALS is approximately 2 cases per 100,000 persons per year, while the prevalence is estimated at 6-9 cases per 100,000 people [133]. Some patients with familial ALS have mutations in the superoxide dismutase 1 (SOD1) gene, which codes for Zn/Cu-superoxide dismutase (SOD1)[134]. Mutant SOD1 proteins exhibit misfolding, increased exposure to copper in the active site, enhanced binding capacity, oligomerization into increasingly higher molecular weights aggregates, and the eventual formation of protein inclusion bodies that are selectively toxic to motor neurons [135]. Furthermore, ALS-associated SOD1 mutants display increased denitrosylase activity, leading to a reduction in GSNO levels, and, consequently, GSNO-induced protein SNO *in vitro* and *in vivo* [136]. In addition, mitochondrial vacuolization and the levels of protein SNO were reduced in the spinal cords of mutant SOD1 transgenic mice, suggesting that ALS-associated mutations in SOD1 enhance its denitrosylase activity and reduces the SNO of mitochondrial proteins, leading to mitochondrial dysfunction, and, ultimately, ALS-related pathology.

Protein disulfide isomerases (PDIs) comprise a class of endoplasmic reticulum-resident enzymes and molecular chaperones whose primary function is to catalyze the formation, breakage, and rearrangement of disulfide bonds in nascent peptide chains. PDI dysfunction is associated with neurodegenerative diseases, including AD, PD, HD, and ALS [137]. PDI was reported to be over-nitrosylated in the spinal cords of SOD1 mutant (G93A) mice and patients with sporadic ALS, which reflects the disease process [138, 139]. SNO of PDI inhibits its activity, triggering mutant SOD1 aggregation and increasing neuronal cell death [138]. In addition, treatment with N-nitro-L-arginine, a NOS inhibitor, reduces the nitrosylation of PDI and the formation of mutant SOD1 aggregates [139]. Combined, these observations show that the denitrosylation of PDI can serve as a therapeutic target for ALS. SOD1 proteins are present in neurons as a mixture of metal-free monomers and dimers (apoSOD1) and partially or fully metalized dimers (holoSOD1) [140]. It was shown that PDI reduces SOD1 monomerization and multimerization and decreases the aggregation of mutant SOD1, whereas an increase in the levels of SNO-PDI leads to incorrect disulfide cross-linking of immature, misfolded mutant SOD1 proteins, which may lead to their aggregation [138]. Partially or fully metal-depleted apoSOD1 protein is thought to be the most likely precursor for ALS [141]. Misfolded SOD1 induces a conformational change in Bcl-2, leading to the exposure of its toxic BH3 structural domain, which triggers cytochrome c release and, ultimately, apoptosis [142]. In addition, misfolded SOD1/Bcl-2 interaction reduces mitochondrial membrane permeability to ADP by directly inhibiting the mitochondrial outer membrane protein VDAC1, which regulates mitochondrial ATP production and export [143]. In motoneurons of presymptomatic G93A mice, this toxic conformation of Bcl-2, triggered by misfolded SOD1, leads to bioenergetic defects, elevated ROS levels, and dysregulation of calcium homeostasis [142]. There is increasing evidence that the levels of S-nitrosylated PDI are increased in the brains of patients with sporadic ALS, PD and AD. Accordingly, targeting PDI denitrosylation may represent a therapeutic strategy for many sporadic cases of these condition.

### Epilepsy

Epilepsy is a chronic non-communicable disease of the brain that affects approximately 70 million people worldwide [144]. The disease is characterized by recurrent seizures. NO has both an anti- and pro-convulsant effect on epilepsy [145], according to the results of studies that have employed multiple NOS inhibitors and NO donors. Previous work has shown that nNOS and NMDA receptor subtype 2B (NMDANR2B) are overexpressed in neocortical dysplasia-associated epilepsy [146]. In addition, studies have shown that the levels of nitrosylated NMDAR are significantly increased in acute seizure and chronic epilepsy [147]. nNOS interacts with NMDARs on the cell membranes via postsynaptic density protein 95 (PSD95), which is essential for the production of NO. Under pathological conditions, NMDAR-mediated excitotoxicity can lead to high Ca^2+^ influx. This generates excess NO, which, in turn, can lead to the SNO of NMDAR, and consequently, reduced receptor hyperactivation [5]. Thus, disrupting the NMDA/PSD95/nNOS axis exerts a protective effect in epilepsy. Notably, the overactivation of NMDARs leads to abnormal increases in intracellular Ca^2+^ concentrations and mitochondrial Ca^2+^ overload. VDAC is a major permeability pathway for mitochondrial Ca^2+^ influx [148]. Ca^2+^ overload leads to mitochondrial damage, the production of ROS and RNS, and alterations in mitochondrial membrane potential, prompting protein nitrosylation, followed by the release of cytochrome C, and, finally, neuronal death. Although it has been documented that VDAC plays an important role in epilepsy [149], whether there is a direct link between nitrosylated VDAC and epilepsy is unclear and merits investigation. Persistent status epilepticus results in reduced Drp1 phosphorylation and the inhibition of mitochondrial fission, leading to neuronal death [150]. Given that the abnormal SNO of Drp1 can increase its enzymatic activity, leading to excessive mitochondrial fission [40], it is particularly important to explore whether the SNO of Drp1 also plays a role in epilepsy. Status epilepticus decreases the level of Drp1 SNO and leads to mitochondrial elongation, leading to neuronal death independently of Trx1 activity [41]. In summary, there is evidence to suggest that NO-mediated SNO may play an important role in the pathology of epilepsy.

### Therapeutic approaches

In humnas, several pathways are involved in protein thiol nitrosylation and denitrosylation. SNO is influenced by a variety of factors, including intracellular redox status, NO levels, and specific reaction sites, and can both protect and impair human health. Therefore, an in-depth understanding of protein SNO and its effects may lead to novel therapeutic approaches for the treatment or prevention of the above-mentioned diseases.

The pharmacological treatment of PD is still mostly reliant on levodopa, a drug that has significant side effects, including dyskinesia and noteworthy cognitive impairment. Consequently, alternatives to levodopa are being actively investigated, including plant-derived natural antioxidants such as curcumin and vitamin C, both of which have neuroprotective effects. Plant polyphenols (such as resveratrol), have strong antioxidant properties. They can influence the redox environment; attenuate oxidative stress, mitochondrial dysfunction, and chronic inflammation; and exert marked neuroprotection effects. The efficacy of resveratrol in AD, stroke and cognitive dysfunction has been demonstrated in clinical trials [151]. The SNO of proteins associated with synaptic function, metabolism, and AD pathology is increased in the early stages of neurodegeneration. In the clinic, neurodegenerative diseases can be prevented or treated by controlling NO production through drug delivery or the modulation of NOS activity. Aberrant NO production can result in abnormal SNO and oxidative phosphorylation of key enzymes of the tricarboxylic acid (TCA) cycle, leading to impaired cerebral energy metabolism and, consequently, neurodegeneration. Therefore, strategies can be adopted to improve mitochondrial function, thereby preventing, or controlling neurological diseases. In addition, by inhibiting the excessive release of α-synuclein, which oligomerizes, aggregates, and deposits in the cytoplasm, Parkin SNO can be reduced, and cells can be protected from extracellular α-synuclein oligomer-induced toxicity, thus contributing to the development of new therapies for PD.

In addition, nutritional dietary interventions can be employed to regulate NO levels. Increasing tyrosine contents through dietary supplements may help reduce the negative effects of inflammation-related BH4 inactivation on the efficiency of dopamine and NO synthesis and ameliorate neurological disorders. Increasing amino acid, polyphenol, and unsaturated fatty acid intake through dietary supplementation may help reduce the negative effects of inflammation and oxidative stress and NO synthesis efficiency, thus ameliorating neurological disorders [152].

For treatment, it is important to pay attention to drug dosage, and be particularly aware of the possibility of stimulation at low doses and inhibition at high doses. As mentioned in the previous section, the level of nitrosylation of proteins under physiological conditions is in dynamic equilibrium, mediated by nitrosylation and denitrosylation. Additionally, the effect of NO-induced SNO depends on the SNO levels of a number of proteins. We therefore hypothesize that there may also be a dose-response relationship for substances that affect SNO levels. This highlights the importance of selecting the appropriate dosage to maximize positive and minimize adverse effects of potential treatments.

## Conclusions

Protein SNO is a recently identified protein post-translational modification that plays an important role in the control and intervention of neurodegenerative diseases. In this review, we highlighted the role of S-nitrosylated proteins in mitochondrial biogenesis, mitochondrial dynamics, and mitophagy, focusing on their association with neurological disorders. With recent advances in SNO proteomic research, we expect to identify an increasing number of SNO proteins that directly or indirectly regulate mitochondrial function. Interestingly, in vitro studies, interfering with only a few or even a single key nitrosylated protein, which plays an important role in neurological disorders, can alleviate mitochondrial dysfunction and protects neurons from damage. Therefore, identifying and exploring the roles of the key SNO proteins that contribute to mitochondrial damage and neuronal injury in each neurological disease is critical to the understanding of the pathophysiological role of protein SNO in these diseases. Studies targeting mitochondrial biogenesis, mitochondrial dynamics, and mitophagy dysregulation in neurological disorders and those aimed at reducing aberrant protein SNO may lead to novel therapeutic options. Although more than 3000 proteins have been identified as being SNO-modifiable outstanding questions remain to be explored in vivo, such as the selection of potential targets and SNO sites, and whether different sites have the same effect when nitrosylated. Moreover, the molecular reaction mechanism of denitrosylation, its role and its relationship with nitrosylation remain unclear. Meanwhile, the level of nitrosylation in vivo and in different tissues determines the different physiological roles it plays. Improving our understanding of the protein nitrosylation network requires a comprehensive and systematic analysis using an interdisciplinary approach that integrates insights from biochemistry, molecular biology, neurology, and pharmacology, aiming to provide new solutions and directions for the clinical control and treatment of a variety of neurological diseases.
